# Imaging Recommendations for Diagnosis and Management of Primary Parathyroid Pathologies: A Comprehensive Review

**DOI:** 10.3390/cancers16142593

**Published:** 2024-07-19

**Authors:** Nivedita Chakrabarty, Abhishek Mahajan, Sandip Basu, Anil K. D’Cruz

**Affiliations:** 1 Department of Radiodiagnosis, Advanced Centre for Treatment, Research and Education in Cancer (ACTREC), Tata Memorial Centre, Homi Bhabha National Institute (HBNI), Parel, Mumbai 400012, Maharashtra, India; dr.niveditachakrabarty@gmail.com; 2 Department of Imaging, The Clatterbridge Cancer Centre NHS Foundation Trust, 65 Pembroke Place, Liverpool L7 8YA, UK; 3 Faculty of Health and Life Sciences, University of Liverpool, Liverpool L69 3BX, UK; 4 Radiation Medicine Centre, Bhabha Atomic Research Centre, Tata Memorial Hospital Annexe, Homi Bhabha National Institute (HBNI), Parel, Mumbai 400012, Maharashtra, India; drsandipb@gmail.com; 5 Apollo Hospitals, Navi Mumbai 400614, Maharashtra, India; docdcruz@gmail.com; 6 Foundation of Head Neck Oncology, Mumbai 400012, Maharashtra, India; 7 Union International Cancer Control (UICC), 1202 Geneva, Switzerland

**Keywords:** primary hyperparathyroidism, parathyroid adenoma, multiglandular parathyroid disease, parathyroid carcinoma, US, 4DCT, ^99m^Tc-sestamibi, 4DMRI, ASCO

## Abstract

**Simple Summary:**

Parathyroid glands are four in number and usually located adjacent to the thyroid gland, however, variations in location and number can exist. Parathyroid glands produce and release parathyroid hormone (PTH) important for maintaining blood calcium levels. In primary hyperparathyroidism (PHPT), there is increased secretion of PTH with resultant elevated blood calcium levels due to causes within the parathyroid gland, such as a single or multiple tumours which could be benign or malignant. The role of imaging is to locate abnormal parathyroid gland including its presence in unusual location if any, localise tumour within the parathyroid gland and plan surgical approach. Additional role of imaging in a suspected case of parathyroid malignancy is to look for any distant spread. There is emerging evidence to suggest that incidental detection of enlarged parathyroid glands on routine CT by the radiologist, can lead to early diagnosis of PHPT before obvious symptoms and signs develop. In this article, we have described the various imaging modalites available for evaluation of various types of parathyroid tumours, along with their imaging appearances and advantages and disadvantages. In addition, we have prepared a flowchart for guiding management decisions for parathyroid tumours.

**Abstract:**

Parathyroid pathologies are suspected based on the biochemical alterations and clinical manifestations, and the predominant roles of imaging in primary hyperparathyroidism are localisation of tumour within parathyroid glands, surgical planning, and to look for any ectopic parathyroid tissue in the setting of recurrent disease. This article provides a comprehensive review of embryology and anatomical variations of parathyroid glands and their clinical relevance, surgical anatomy of parathyroid glands, differentiation between multiglandular parathyroid disease, solitary adenoma, atypical parathyroid tumour, and parathyroid carcinoma. The roles, advantages and limitations of ultrasound, four-dimensional computed tomography (4DCT), radiolabelled technetium-99 (^99m^Tc) sestamibi or dual tracer ^99m^Tc pertechnetate and ^99m^Tc-sestamibi with or without single photon emission computed tomography (SPECT) or SPECT/CT, dynamic enhanced magnetic resonance imaging (4DMRI), and fluoro-choline positron emission tomography (^18^F-FCH PET) or [^11^C] Methionine (^11^C -MET) PET in the management of parathyroid lesions have been extensively discussed in this article. The role of fluorodeoxyglucose PET (FDG-PET) has also been elucidated in this article. Management guidelines for parathyroid carcinoma proposed by the American Society of Clinical Oncology (ASCO) have also been described. An algorithm for management of parathyroid lesions has been provided at the end to serve as a quick reference guide for radiologists, clinicians and surgeons.

## 1. Introduction

Parathyroid glands synthesize and release parathyroid hormone (PTH) essential for regulation of serum calcium levels. Primary hyperparathyroidism (PHPT) is the third most common endocrine pathology in which there is increased secretion of PTH with resultant elevated serum calcium levels due to causes intrinsic to parathyroid gland, such as a solitary adenoma (commonest cause with 80–85% incidence), multiglandular parathyroid disease (previously known as hyperplasia, with 10–15% incidence), atypical parathyroid tumour (previously known as atypical adenoma, with 1.2–1.3% incidence), and parathyroid carcinoma (0.1–5% incidence) [1,2,3]. PHPT is more commonly seen in women more than 50 years of age and can be sporadic (most common, with 95% incidence), associated with radiation exposure or sarcoidosis, or seen in association with hereditary and genetic syndromes, for example, hyperparathyroidism jaw-tumour syndrome (HPJT), multiple endocrine neoplasia syndromes type 1, type 2A and 4, and isolated familial hyperparathyroidism [1,4,5,6,7,8,9,10]. Parathyroid carcinoma has equal gender incidence [5].

It is not possible to differentiate between various benign causes of PHPT based on the elevated levels of serum calcium and PTH; moreover, PHPT can also be normocalcemic [1,11,12]. Parathyroid carcinomas may be suspected with serum calcium > 14–15 mg/dL and markedly elevated levels of PTH; however, <10% of parathyroid carcinomas can be hormonally non-functional and can only be suspected based on clinical examination findings [5,11,13,14,15].

Imaging cannot differentiate parathyroid adenoma from carcinoma, and diagnosis is most often accomplished after parathyroidectomy [5]. The predominant role of imaging is to locate abnormal parathyroid gland, including the presence of ectopic gland, if any, localise lesions within parathyroid gland, and plan the surgical approach in case of PHPT and when there is clinical suspicion of non-functioning parathyroid carcinoma. An additional role of imaging in a suspected case of parathyroid carcinoma is to look for any distant metastases. There is emerging evidence to suggest that biochemical screening, based on opportunistic detection of enlarged parathyroid glands on routine CT by the radiologist, can lead to early diagnosis of primary hyperparathyroidism before frank manifestations develop [16,17].

Imaging plays a crucial role in recurrent disease. Morphological information, including anatomic localisation and the extent of the parathyroid lesion, is provided by ultrasound (US), including the recent contrast-enhanced US (CEUS), four-dimensional computed tomography (4DCT) and dynamic enhanced magnetic resonance imaging (4DMRI), whereas functional information is provided by radiolabelled Technetium-99 (^99m^Tc) sestamibi or dual tracer ^99m^Tc pertechnetate and ^99m^Tc-sestamibi with or without single photon emission computed tomography (SPECT) or SPECT/CT and fluoro-choline positron emission tomography (^18^F-FCH PET or [^11^C]Methionine (11C-MET) PET [1,18,19]. Hybrid imaging such as ^18^F-FCH PET/4DCT and ^18^F-FCH PET/MRI can capture advantages of both structural and functional imaging [1]. Surgical removal is the mainstay treatment for parathyroid lesions, and precise localisation of abnormal parathyroid glands/parathyroid lesions on imaging is crucial to perform focussed surgeries such as minimally invasive parathyroidectomy (MIP) [4,20]. 

In this review article, we have comprehensively covered all aspects of parathyroid gland imaging, including anatomy, embryology, variations and their clinical significance, the role of various imaging modalities, their advantages and limitations, and the clinical, laboratory and imaging features of various parathyroid lesions highlighting key differentiating features. We have also provided an algorithm for the management of parathyroid lesions at the end to serve as a reference guide for radiologists, clinicians and surgeons.

## 2. Anatomy of Parathyroid Glands

In this section, we have described the embryology, anatomical variations, and surgical anatomy of parathyroid glands. The clinical significance of embryology and anatomical variations of parathyroid glands have also been described in this section.

### 2.1. Embryology

There are most commonly four parathyroid glands; one superior and one inferior on either side [1,4,20]. The parathyroid glands originate at 5–6 weeks of gestation, from the endoderm epithelial cells of the pharyngeal pouches [21]. At week 7 of gestation, they begin to migrate along with the thyroid and thymus inferiorly and medially [21].

Superior parathyroid glands are derived from the fourth branchial pouch and are associated with lateral thyroid anlage [21]. They attach to the inferiorly migrating thyroid gland and are closely related to the posterolateral aspect of the respective thyroid lobes [21]. In >80% of the cases, the final positions of superior parathyroid glands are posterolateral to the thyroid gland superior pole at the level of cricothyroid articulation [20,21].

Inferior parathyroid glands are derived from the third branchial pouch sharing a common origin with the thymus gland; inferior parathyroid glands arise from the dorsal wing, and the thymus gland arises from the ventral wing of the third branchial pouch [4,21]. The inferior parathyroid glands descend caudally and medially along with the thymus and are most commonly located at the posterolateral aspect of the inferior pole of the thyroid gland or within 1–2 cm [21,22,23].

Parathyroid glands are composed of chief cells and oxyphilic cells, fatty tissue, and fibrovascular stroma [24].

### 2.2. Anatomical Variations

*Supernumerary glands:* Supernumerary glands are seen in 13% of the population; 2–3 glands are seen in 10% of the population, 5 glands in 5%, and 6 glands in 0.2% [1,21,25]. The maximum number of supernumerary glands reported in the literature was 12 [25,26].

*Ectopic parathyroid gland:* Superior parathyroid glands are more consistent in their location owing to their shorter course of embryologic migration [21]. In approximately 4% of cases, superior parathyroid glands can be located posterior to the mid-pole of thyroid lobes, in 2% cases at the level of the upper pole, and in less than 1% of cases, they can be located above the upper pole of the thyroid gland [4,21]. Superior parathyroid glands can be ectopically located in the tracheoesophageal groove, retroesophageal (1%), retropharyngeal (1%), posterior mediastinum, and intrathyroidal locations (0.2%) [4,21,26,27].

The inferior parathyroid glands are more prone to anatomical variations due to their longer course of migration and can be located anywhere up to the superior border of the pericardium [20,21]. If an inferior parathyroid gland fails to descend with the thymus, it can be located close to the carotid bifurcation or within the ectopic thymic remnant [21]. In 2% cases, it can be located close to thymus in the anterior mediastinum, and the aortopulmonary window is another ectopic location [4,28]. Rarely, it can be located ectopically within the thyroid or cranially to the superior parathyroid gland [4,21]. Ectopic parathyroid glands can also be located in the submandibular and parotid glands [29,30,31,32,33].

### 2.3. Surgical Anatomy

The plane of the recurrent laryngeal nerve (RLN) close to the tracheoesophageal groove differentiates between superior and inferior parathyroid glands, as the superior parathyroid gland lies posterior to the RLN, and the inferior parathyroid gland lies anterior to it [20,21]. However, operative landmarks are altered in the presence of a non-recurrent laryngeal nerve [20]. Eighty percent of superior parathyroid glands are located approximately 1 cm cranially to the intersection of the RLN and inferior thyroid artery at the level of cricothyroid articulation, and within 2 cm posteriorly to the thyroid gland [21,26,34]. In >80% cases, once the surgeons have located one side of a superior parathyroid gland, they will be able to locate the other side, as they have mirror symmetry. The inferior parathyroid glands have mirror symmetry in >70% of cases [21,26,35]. In 76–86% of cases, parathyroid glands are supplied by the inferior thyroid artery [36].

### 2.4. Clinical Significance

Knowledge of the embryology and anatomy of parathyroid glands is essential to identify lesions of the parathyroid glands on imaging. Bilateral neck exploration (BNE) and minimally invasive parathyroidectomy (MIP) are the two operations usually performed for PHPT. BNE allows visualisation of all the parathyroid glands with a single midline 2.5 cm incision and has a brilliant long-term cure rate (>95%), but carries the risk of injuring the bilateral RLN with resultant increased morbidity and longer hospital stay [20]. On the other hand, MIP entails removal of only the diseased gland with a small incision, with lesser morbidity and hospital stay, but requires precise pre-operative localisation of the diseased gland on imaging [20].

In-depth knowledge of anatomical variations helps to locate ectopic parathyroid glands on imaging, which in turn helps in surgical planning. Failure to locate ectopic glands can result in failed surgical explorations and persistent PHPT [20].

A non-recurrent laryngeal nerve is more likely to be injured if the surgeons are unaware of its presence pre-operatively on imaging. Hence, an aberrant right subclavian artery and right-sided aortic arch with aberrant left subclavian artery should be mentioned on pre-operative imaging, as they exhibit non-recurrent right and left laryngeal nerves, respectively [20,34].

## 3. Clinical Manifestations and Laboratory Investigations of Parathyroid Lesions

Approximately 70–80% of the patients with benign PHPT are asymptomatic and detected incidentally due to elevated serum calcium levels, and the remaining patients present with symptoms and/or signs of PHPT, such as nephrolithiasis, nephrocalcinosis, hypercalciuria, osteoporosis, osteitis fibrosa cystica, fragility fractures, hyperlipidaemia, diabetes, constipation, ileus, peptic ulcers, pancreatitis, and/or symptoms of hypercalcemia; fatigue, weakness, depression, anxiety, and cognitive impairment [10,37,38,39]. Asymptomatic patients can still have nephrolithiasis/nephrocalcinosis and decreased trabecular/cortical bone density [10]. Normocalcemic variants of primary HPT can also occur, of which some may show reduced bone mineral density [10,40,41,42,43,44,45]. Normohormonal PHPT refers to patients with a normal PTH and elevated calcium levels, and such patients have a greater propensity for multigland disease [46]. Five percent of PHPT cases have associated hereditary syndromes and present before 30 years of age with familial hypercalcaemia, MEN syndromes, skin lesions, and HPJT. Familial hypocalciuric hypercalcaemia (FHH) syndrome should be suspected when there is hypocalciuria along with hypercalcaemia [37,42,47,48,49,50,51,52,53,54,55,56,57].

Parathyroid carcinoma can be hormonally functional or non-functional. Clinical presentation of hormonally functional parathyroid carcinoma is similar to that of PHPT and should be suspected when there are more severe symptoms and multiple system involvement, particularly simultaneous skeletal (bones including jaw) and renal involvement due to a more profound hypercalcemia, and familial syndromes [5,10,37,38,39,58,59,60,61,62,63,64,65,66,67,68,69,70].

Laboratory values of PTH and serum calcium cannot differentiate between various causes of PHPT, unless serum calcium is more than 14 mg/dL or PTH is more than three times the upper limit of normal, in which case parathyroid carcinoma should be suspected [5,11,13]. However, <10% of parathyroid carcinomas can be hormonally non-functional and suspected based on palpable neck nodes, hoarseness of voice due to recurrent laryngeal nerve (RLN) involvement, and metastatic disease [1,5,12,14,15]. Of patients with parathyroid carcinoma, 6–30% present with lymph node metastasis, and 10–30% present with metastases to lungs, liver or bones at the time of presentation [5,10,58,59,60,61,62,71].

The current 2022 World Health Organization (WHO) classification of parathyroid tumours endorses usage of the terminology “multiglandular parathyroid disease (MGD)” in place of hyperplasia in the setting of PHPT, and has replaced the terminology “atypical adenoma” with “atypical parathyroid tumour” [3]. For the diagnosis of parathyroid carcinoma, one of the following findings is essential; angioinvasion, lymphatic invasion, perineural invasion, invasion into adjacent structures locally, or, presence of metastasis [3]. Atypical parathyroid adenoma is a borderline tumour of uncertain malignant potential which shares some histopathological findings of parathyroid carcinomas, such as band forming fibrosis, increased mitotic activity, and presence of tumour cells within a thickened capsule; however, definitive diagnostic findings of malignancy in the form of invasion and metastasis are absent [3,10,51,72,73,74,75,76].

FNAB of a suspected parathyroid carcinoma is not recommended, as it cannot differentiate adenoma from carcinoma, and also, because it carries the risk of tumour seeding and upstaging the disease [5,77,78]. Diagnosis of parathyroid carcinoma is most often accomplished after parathyroidectomy [5]. While evaluating a recurrent disease in a known case of parathyroid carcinoma, a preoperative biopsy may be performed.

## 4. Imaging of Parathyroid Lesions

The goals of imaging are to locate the abnormal parathyroid gland; orthotopic or ectopic location, localise lesion within the parathyroid gland and help in surgical planning, and asses for invasiveness, enlarged neck nodes and distant metastasis in a suspected case of parathyroid carcinoma [79]. An additional role of imaging, as already specified, is to detect enlarged parathyroid glands on routine CT examinations, so that biochemical screening can be performed based on the CT findings and patients with asymptomatic PHPT can be identified early, thus narrowing the gap between diagnosis and treatment initiation before frank manifestations develop [16,17]. Posttreatment imaging should be performed when there is a suspicion of recurrence or elevated PTH or hypercalcemia [5]. Studies have shown that normal parathyroid gland can be identified on US mainly at the lower pole of the thyroid and in the infra-thyroid location as an oval-shaped homogeneously hyperechoic structure [80,81].

Various imaging modalities for evaluation of parathyroid gland lesions include neck US including CEUS, single photon scintigraphy with radiolabelled Technetium-99 sestamibi or dual tracer ^99m^Tc pertechnetate and ^99m^Tc-sestamibi with or without SPECT or SPECT/CT, 4DCT, 4DMRI, and fluoro-choline positron emission tomography (^18^F-FCH PET) or [^11^C]Methionine (11C-MET) PET [1,18,19]. The roles of each of these imaging modalities, along with their advantages and limitations, have been extensively described in this section. Hybrid imaging, such as ^18^F-FCH PET/4DCT and ^18^F-FCH PET/MRI for evaluation of parathyroid lesions, has also been elucidated in this section. In addition, the role of fluorodeoxyglucose PET (FDG-PET) has also been described in this section.

### 4.1. Neck Ultrasound and Contrast-Enhanced Ultrasound

**Figure 1:**
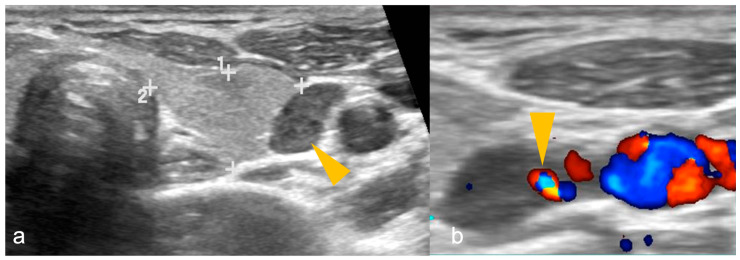
(**a**,**b**): Parathyroid adenoma on ultrasonography in a patient with primary hyperparathyroidism. (**a**) A well-defined oval-shaped homogeneously hypoechoic lesion (arrowhead) lateral to the left lobe of thyroid gland (shown by 1, 2 and + sign). (**b**) Colour Doppler image shows feeding vessel sign (arrowhead). Imaging findings are suggestive of parathyroid adenoma.

The patient is scanned with mild neck extension using a linear array high-frequency probe (7.5–15 MHz) in transverse and longitudinal planes with a special focus behind the thyroid gland medial to the carotid and jugular vessels where the parathyroid glands are usually located [18]. The neck should be scanned from the carotid bifurcation to sternal notch and the paratracheal spaces, carotid-jugular axis, and thyroid gland should be included [79]. PA is a well-circumscribed, oval- or oblong-shaped, hypoechoic lesion compared to the adjacent thyroid gland, located posterior (more commonly), anterior or lateral to the thyroid at the superior/inferior polar regions, having an echogenic capsule, and shows an enlarged feeding inferior thyroidal artery (feeding vessel sign) on colour Doppler with a low resistive index on spectral Doppler [1,18]. Internal heterogeneity due to fat, calcifications or haemorrhage can be seen [1]. US of a PA is shown in Figure 1.

Multiple adenomas can be seen when associated with MEN 1 syndrome. MGD shows bilaterally enlarged (may be asymmetric) homogeneous glands adjacent to the thyroid at the polar regions. Parathyroid carcinoma can be seen as an inhomogeneous hypoechoic lesion epicentred adjacent to the thyroid with hypervascularity on colour Doppler and the presence of cystic degeneration [1,82,83]. US features in favour of parathyroid carcinoma include large size (>3 cm), lobulated margins, central and peripheral vascularity, microcalcifications, and the presence of metastatic neck nodes [5,82,83,84,85]. Intraoperative US can guide the surgical approach, as suggested by the American Head and Neck Society (AHNS) Endocrine Section guidelines [1,86].

CEUS can help differentiate PA from MGD, as PA shows early peripheral enhancement and central washout in the delayed phase, whereas MGD show intense homogeneous enhancement [82,83,86,87,88]. Shear wave elastography can help differentiate PA from surrounding thyroid tissue by demonstrating significantly lower elasticity than thyroid tissue [89]. In addition, US is the optimal modality for assessing the thyroid gland for concurrent thyroid pathology.

### 4.2. Dual Phase Technetium-99 Sestamibi, and Dual Tracer Technetium-99 Pertechnetate and Technetium-99 -Sestamibi Scintigraphy, with or without Single Photon Emission Computed Tomography or Single Photon Emission Computed Tomography/Computed Tomography

The principle behind ^99m^Tc Sestamibi imaging is the increased accumulation of ^99m^Tc sestamibi in hyperfunctioning parathyroid gland due to abundant mitochondria within the oxyphil cells of parathyroid gland [9]. A typical protocol for ^99m^Tc Sestamibi imaging is shown in Table 1 [9].

**Figure 2:**
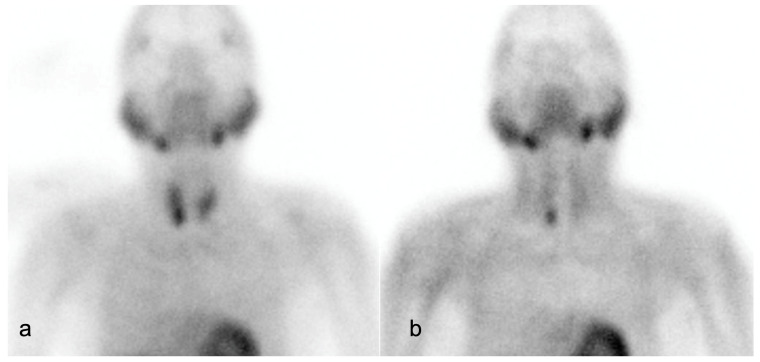
A 61-year-old male with recurrent renal stones presented with lower back ache. Serum parathyroid hormone: 2231.3 pg/mL. (**a**) Early image (performed at 15 min post-^99m^Tc- methoxyisobutylisonitrile [MIBI] injection, planar static imaging of the cervical and thoracic area in the anterior view): Homogenous tracer uptake in both lobes of the thyroid gland, with a focus of increased radiotracer accumulation seen in the region of inferior pole of right lobe of thyroid. (**b**) Delayed image (performed 120 min after the ^99m^Tc MIBI injection): Persistent focal moderately increased tracer retention in the region of inferior pole of right lobe of thyroid gland, with washout of tracer from the rest of the thyroid gland.

**Figure 3:**
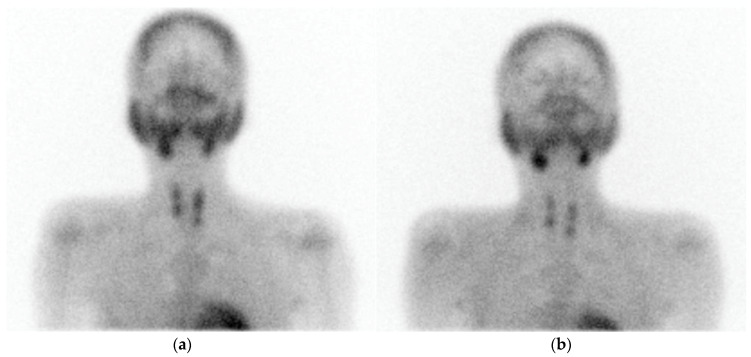
A 33-year-old male presented with joint pain while walking, with pain in right more than left hip, along with back and knee pain. Serum parathyroid hormone: >2500 pg/mL. Computed tomography (CT) of hip joints revealed diffuse osteopenia, multiple lytic lesions (brown tumour) in lumbar vertebrae and pelvic bones. (**a**) Early static views (performed at 15 min post ^99m^Tc-methoxyisobutylisonitrile [MIBI] injection) show tracer uptake in neck coinciding with both lobes of the thyroid gland, with focal uptake noted over upper and lower poles of both thyroid lobes. (**b**) Delayed image at 3 h post ^99m^Tc-MIBI injection shows clearance of tracer from both lobes of thyroid, except focal tracer retention noted bilaterally over upper and lower poles of both thyroid lobes.

On ^99m^Tc Sestamibi, PA shows focal increased radiotracer uptake near the superior/inferior polar region of the thyroid in the early phase, with persistent uptake in the delayed phase. It has advantages over US in detecting ectopic and far-posterior lesions [90]. The dual phase allows differentiation of PA and MGD from the normal thyroid tissue, as delayed wash-out is seen in PA and MGD, and early wash-out is seen in thyroid tissue, while both show uptake [4]. Figure 2 and Figure 3 show parathyroid adenoma and multiglandular parathyroid disease, respectively, on ^99m^Tc Sestamibi.

Impression: Dual-phase (early and delayed) ^99m^Tc-MIBI parathyroid scintigraphy demonstrates parathyroid adenoma located inferior to the lower pole of right lobe of thyroid gland.

Impression: Dual-phase (early and delayed) ^99m^Tc-MIBI parathyroid scintigraphy demonstrates features suggestive of parathyroid hyperplasia.

False positive results can occur in the presence of thyroid nodules and neck nodal metastases [90].

In some cases, tracer may rapidly wash out from parathyroid or be retained by thyroid/solid thyroid nodules, and such cases may require the dual-tracer method using ^99m^Tc pertechnetate and ^99m^Tc-sestamibi scintigraphy [9,18]. In addition, this single-phase dual-isotope technique has increased sensitivity for detecting MGD as compared to single-isotope dual-phase scans [90].

**Figure 4:**
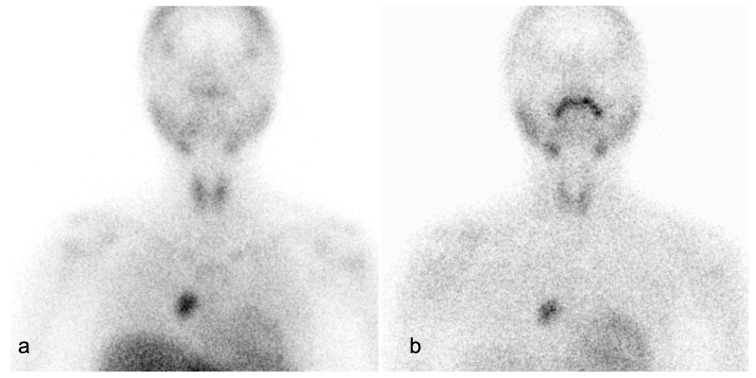
A 23-year-old female with pain in bilateral hip, knee and elbow joints associated with weakness in walking. Ultrasonography (USG) was normal. Computed tomography (CT) showed a 6.8 × 10.7 × 15.4 cm enhancing lesion in suprasternal space posteriorly, abutting the left infrahyoid strap muscle between the brachiocephalic trunk and left common carotid artery (CCA), suspicious for ectopic parathyroid adenoma in suprasternal space. (**a**) Early static views show areas of increased tracer uptake in both lobes of the thyroid, with focus of abnormal tracer concentration in the superior mediastinum at the right paracardiac region. (**b**) Delayed image at 3 h post-injection shows almost complete washout of the tracer from both lobes of the thyroid, with persistent tracer uptake at the right paracardiac region.

Scintigraphy can aid in preoperative identification of hyperfunctioning parathyroid glands in typical as well as in ectopic locations (Figure 4) [9].

Impression: Ectopic parathyroid in mediastinum at the right paracardiac region.

SPECT images, when fused with CT, provide better anatomic localisation compared to planar images [9,91].

^99m^Tc Sestamibi scan may also be used pre-operatively to detect the parathyroid gland with the lowest uptake, which may be partially autografted or preserved [9]. It also plays a role in the recurrent setting to localise the hyperfunctioning gland, prior to second surgery [9].

### 4.3. Four-Dimensional Computed Tomography

Four-dimensional computed tomography (4DCT) is a multiphasic multiplanar dynamic contrast-enhanced CT of the parathyroid gland in which usually a three-phase study is performed; non-contrast, arterial phase and delayed venous phase [1,20]. Important information that a surgeon expects from a radiologist on a 4DCT includes: a. number of lesions, b. size of the lesion, c. location of the parathyroid lesion with respect to surgical landmarks such as cricoid cartilage, tracheoesophageal groove, d. presence or absence of ectopic parathyroid tissue, e. associated thyroid abnormalities (if present), and f. arterial anomalies associated with a nonrecurrent laryngeal nerve, for example, aberrant right subclavian artery or right-sided aortic arch with aberrant left subclavian artery [20]. A typical parathyroid adenoma is usually hypodense to thyroid on non-contrast scan, hyper-enhancing in the arterial phase with washout in the venous phase, usually located posterior to thyroid at the superior/inferior polar regions [20]. PA may show an enlarged feeding inferior thyroidal artery (polar vessel sign) in the arterial phase [1]. For parathyroid lesions that are isodense to thyroid in the arterial and venous phases, dual-energy CT 4DCT can help in differentiating the parathyroid lesion from thyroid tissue using non-contrast 40-keV virtual monoenergetic images [92].

One of the studies showed that an irregularly shaped parathyroid lesion showing heterogeneity, invasion of surrounding structures, short/long-axis ratio > 0.76, and long axis diameter > 30 mm, had high negative predictive value, and the presence of calcification within the tumour had 100% positive predictive value to diagnose for parathyroid carcinoma on CECT. The presence of metastatic neck nodes should raise suspicion of parathyroid carcinoma [20,85]. A typical 4DCT protocol is shown in Table 2 [1,20].

**Figure 5:**
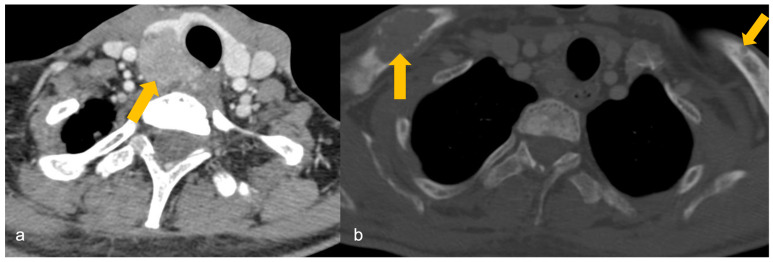
A 32-year-old male with a family history of parathyroid carcinoma presented with elevated serum calcium and parathyroid hormone. (**a**) Heterogeneously enhancing mass (arrow) arising posterior to the right lobe of thyroid gland on contrast-enhanced computed tomography (CECT), infiltrating the thyroid lobe and occupying the right trachea-oesophageal groove, findings suggestive of parathyroid carcinoma. (**b**) Osteolytic lesions in bilateral clavicles (arrows) on CECT, suggestive of biopsy-proven brown tumours.

Imaging cannot differentiate parathyroid adenoma (PA) from carcinoma unless cervical adenopathy or distant metastasis is present to suggest parathyroid carcinoma (Figure 5).

Two scoring systems have been developed and prospectively validated for predicting MGD using 4DCT: a composite multigland disease score calculated from 4DCT imaging findings (number of lesions and maximum diameter of the largest lesion) and the Wisconsin Index (the product of the serum calcium and PTH levels), and a 4DCT multigland disease score obtained by using the CT data alone [93,94]. Both these scoring systems have been found to be valuable in surgical planning by predicting MGD with specificities of 72%, 86%, and 100% for composite MGD scores of ≥4, ≥5, and 6, respectively, and 74% and 88% for 4DCT scores of ≥3 and 4, respectively, in the prospective setting [93,94].

### 4.4. Four-Dimensional Magnetic Resonance Imaging

It is usually a second-line modality for problem-solving in equivocal cases or may be used as an alternative imaging modality in place of 4DCT to avoid radiation dose [1]. One of the studies has shown that dynamic contrast-enhanced MRI has excellent diagnostic performance for preoperative localisation in primary hyperparathyroidism: 92% for single-gland disease and 74% in MGD [95]. Features associated with PA include oval shape with longest to shortest diameter ratio of >2, homogeneous or marbled T2 hyperintensity, fluid fat interface on out-of-phase imaging between thyroid gland and PA (not seen in intrathyroidal PA), and rapid post-contrast enhancement [96]. Studies have shown that a combination of time-to-peak/wash-in/washout using dynamic 4DMRI can help in differentiating PA from neck nodes, as PAs show significantly quicker time-to-peak, higher wash-in, and higher washout compared with neck nodes, and this combination can also help in differentiating PA from thyroid tissue, as PAs show higher peak enhancement, quicker time-to-peak, higher wash-in, and higher washout compared with thyroid tissue [97].

**Figure 6:**
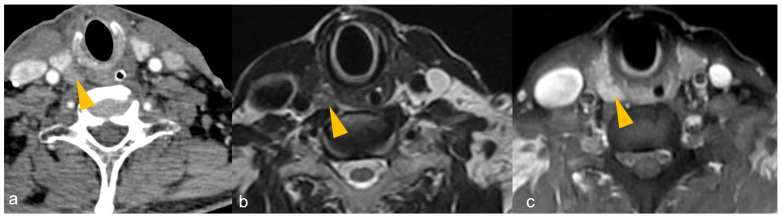
(**a**–**c**): CT and MRI of parathyroid adenoma in a patient with primary hyperparathyroidism. (**a**) Late venous phase 4DCT shows a well defined oval-shaped hypodense lesion (arrowhead) measuring 0.9 × 0.8 cm posterior to the inferior pole of right lobe of thyroid gland, suggestive of parathyroid adenoma. T2WI (**b**) shows intermediate signal intensity of the parathyroid adenoma, which shows intense post-contrast enhancement (arrowhead in (**c**)).

Figure 6 shows CT and MRI of a PA in a patient with PHPT.

### 4.5. Fluoro-Choline Positron Emission Tomography and ^11^C Methionine Positron Emission Tomography

^18^F-FCH is not a parathyroid-specific biomarker but a generalised tracer, taken up by the neoplastic cells [98]. Neoplastic cells with high proliferative rates show increased demand for phospholipid synthesis and hence take up choline [1,99]. PAs show increased choline uptake due to increased lipid-dependent choline kinase activity from elevated PTH secretion [100]. Brown tumours also show increased uptake [101,102]. In comparison to Technetium (99mTc) Sestamibi imaging, ^18^F-FCH PET has better resolution and lesser acquisition time, hence it is accepted as an alternative first-line imaging modality [9,103,104,105].

^11^C-MET PET may serve as a dependable second-line imaging modality to enable MIP, owing to its overall good sensitivity and positive predictive value [19].

### 4.6. Hybrid Imaging with Fluoro-Choline Positron Emission Tomography^/^Four-Dimensional Computed Tomography and Fluoro-Choline Positron Emission Tomography^/^Magnetic Resonance Imaging

Improved structural characterization has been found with both these hybrid imaging techniques [1,106]. ^18^F-FCH PET/MRI is more useful for characterizing parathyroid lesions in the paediatric population and for guiding curative surgeries [107].

### 4.7. Fluorodeoxyglucose Positron Emission Tomography

The role of FDG PET-CECT is to detect distant metastases in a suspected case of parathyroid carcinoma. Additional imaging for metastatic disease is not routinely performed for hyperparathyroidism unless the suspicion for parathyroid carcinoma is high [5,108,109]. For detection of recurrence, FDG PET-CECT should be performed 3–6 months after treatment [5].

Table 3 shows the difference between MGD, solitary adenoma and carcinoma based on clinical and laboratory parameters and imaging [5,10,11,13,18,84,85,92].

Table 4 shows advantages and limitations of various imaging modalities [1,9,18,90,110,111].

## 5. Comparative Studies on Performance of Various Imaging Modalities

Table 5 shows studies comparing the performance of various imaging modalities in the evaluation of PHPT based on a PubMed search covering the last 5 years [112,113,114,115,116,117,118,119,120,121,122,123,124]. Purely imaging studies having comparison between at least two imaging modalities have been incorporated in this table. As shown in Table 5, US and 4DCT can be considered as the first-line imaging modalities for evaluation of PHPT. FCH PET/CT has a greater sensitivity than 4DCT in detecting lesions in PHPT. ^99m^Tc-MIBI scintigraphy could increase the specificity in paediatric patients suspected to have multigland disease on US.

A study by Christakis et al. on parathyroid carcinoma showed accuracies of 80%, 82%, 95% for US, 4DCT and ^99^Tc MIBI SPECT/CT respectively and a combined accuracy of 100% [125].

## 6. Parathyroid Venous Sampling

Parathyroid venous sampling (PVS) or selective venous sampling is an invasive method to localise abnormal parathyroid glands in the setting of PHPT (hypercalcemia and elevated PTH levels). In PVS, abnormal parathyroid glands can be located based on the territory drained by a particular vein/veins and noting the corresponding PTH concentrations [126].

### 6.1. Indications of PVS

1. Inability of non-invasive studies to pre-operatively locate abnormal parathyroid gland [127].
2. Discordant findings between different imaging modalities regarding location of abnormal parathyroid gland [127].
3. Post-surgical persistent hypercalcemia and elevated parathyroid hormone (PTH) levels [126,128].
4. Recurrence of PHPT after prior surgery, with non-visualization or discordant repeat non-invasive imaging findings [126,128].
5. In patients with familial hyperparathyroid syndromes who frequently have MGD [126].

### 6.2. Venous Drainage of Parathyroid Glands

Awareness regarding normal and abnormal venous drainage of normally located and ectopic parathyroid glands is pertinent for the interpretation of PVS results.

The thyroid plexus (formed by the three pairs of thyroid veins) provides the drainage pathway for the parathyroid veins, which subsequently drain inferiorly via the inferior thyroid veins [129]. The superior, middle and inferior thyroid veins drain the superior thyroid pole, mid-thyroid pole and inferior thyroid pole, respectively. Both superior and middle thyroid veins drain into the ipsilateral internal jugular vein (IJV) [130]. The drainage of inferior thyroid veins is commonly into the left brachiocephalic vein, either separately or by forming a common trunk. Less frequently, the right inferior thyroid vein drains directly into the right brachiocephalic vein [129].

Ectopically located parathyroid glands in the mediastinum drain mainly into the thymic vein, and occasionally into the inferior mesenteric vein (IMV) or into the inferior thyroid vein common trunk [131]. Drainage of the left thymic vein is into the antero-inferior aspect of the left brachiocephalic vein in the midline, whereas drainage of the right thymic vein is directly into the superior vena cava (SVC) and cannot be normally catheterised.

### 6.3. Procedure and Interpretation of Parathyroid Venous Sampling

After a standard Seldinger approach from the common femoral vein, a baseline blood sample may be taken from the common iliac vein or the SVC to serve as a baseline control [126]. It is important to selectively catheterise inferior, middle and superior thyroid veins and thymic veins and obtain samples from these sites. Upon selective catheterisation of one of the thyroid veins, a retrograde venogram can help identify the anatomy, facilitating localisation of other desired vessels [126]. When selective sampling is not possible, IJV should be sampled at superior, middle and inferior locations, along with sampling of the left brachiocephalic vein (left side, mid and right side). All the mediastinal veins draining into the inferior left brachiocephalic vein should be catheterised and sampled. Blood samples can also be taken from the unusual sites of right atrium, internal mammary veins, infrarenal inferior vena cava (IVC), suprarenal IVC, and from the hepatic veins, for localisation of metastatic parathyroid carcinoma.

These samples are properly labelled with regard to the locations within the veins from where they were obtained, and are either sent to a laboratory for PTH assays or used for PTH assays performed onsite [126]. A 1.5–2-fold increase in the PTH level from a specific cervical or mediastinal vein, in comparison to a peripheral vein, is considered to be unusually elevated [132,133,134,135]. A super-selective venous sampling study with real-time rapid PTH assay gave a sensitivity and positive predictive value of 86% and 93%, respectively, with a gradient of ≥2 [133].

## 7. Algorithm for Management

**Figure 7:**
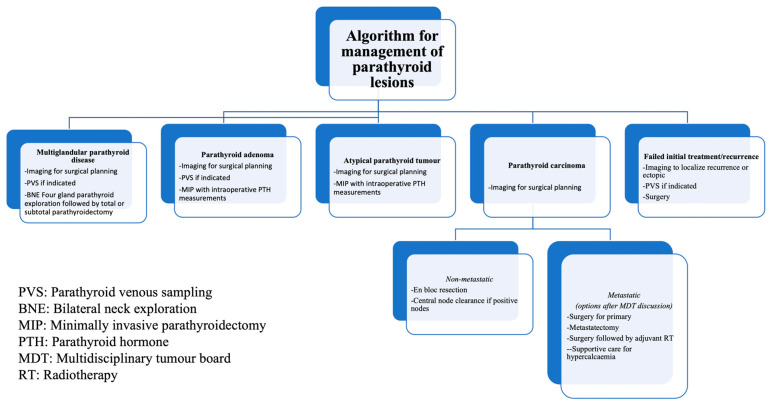
Algorithm for management of parathyroid lesions.

An algorithm for management of parathyroid lesions is shown in the flowchart below (Figure 7).

American Society of Clinical Oncology (ASCO) recommendations are practised for the management of parathyroid carcinomas [5,8]. Parathyroid carcinomas are staged as localised, metastatic, or recurrent instead of using the four-stage system [5]. Surgical removal without capsular disruption to achieve R0 resection (grossly and microscopically negative margins) is the mainstay treatment [5]. Central regional lymph nodal clearance should be performed with suspected nodal involvement [5,13,71]. RLN, though preserved, may be resected if the tumour capsule abuts or invades RLN [5]. Intraoperative PTH levels may return to normal after resection of hormonally active disease; however, persistent elevation may suggest metastatic disease, hence re-exploration should not be performed [5]. Re-exploration with en bloc resection is warranted if postoperative specimen histology is concerning for malignancy or atypia [113,136,137,138,139,140,141]. There is no role for chemotherapy, and no standard radiotherapy exists for parathyroid carcinoma. Decisions for adjuvant RT are to be made in a multidisciplinary tumour board on an individualised basis [5].

## 8. Role of Artificial Intelligence

Artificial intelligence-related research in oncology, mainly using deep learning, has provided an impetus for holistic cancer care, including precision oncology [142,143]. Quite a few studies have been conducted to locate abnormal parathyroid glands and identify abnormalities (mainly adenoma) using machine learning (ML) and deep learning (DL) algorithms on imaging, with or without the combination of clinical and laboratory parameters, and have shown promising results [144]. While most of the studies have been based on parathyroid scintigraphy, one of the studies employed US images [145,146,147,148,149]. One of the studies showed the feasibility of using DL with FCH-PET to detect and localise PHPT [150]. Two studies used radiomic features for identifying PA; one extracted radiomic features from delayed parathyroid SPECT combined with ML, and another correlated radiomic data of 4DCT with pathology-proven PA [151,152].

These studies have shown that artificial intelligence can help in the preoperative identification and localisation of PA and detection of MGD, and large imaging datasets and explainable algorithms can further enhance its utility.

## 9. Conclusions

Ultrasound (US) and 4DCT are typically the first-line imaging modalities for evaluating primary hyperparathyroidism (PHPT). Choline PET offers increased sensitivity for detecting small lesions and improved localisation, though it is more expensive. Technetium-99 sestamibi scans can identify hyperfunctioning parathyroid glands in both orthotopic and ectopic locations but have limited efficacy in cases of multigland disease. Parathyroid venous sampling can be useful when pre-operative imaging results are discordant or in recurrent cases. FDG PET-CECT plays a role in assessing distant metastasis in parathyroid carcinoma.

## Figures and Tables

**Table 1:** Protocol for parathyroid ^99m^Tc sestamibi imaging.

Parameters	Description
Coverage	Entire neck and thorax till base of heart
Phases	Dual phase: at 10–15 min and 90–150 min after intravenous administration of radiopharmaceutical
Image acquisition	- Large field-of-view gamma cameras with low-energy high-resolution collimators. - Additionally, SPECT/CT from skull base to heart base
Energy window	140 ± 10 keV
Matrix size	128 × 128 or 256 × 256 (better)

SPECT: single photon emission computed tomography; CT: computed tomography.

**Table 2:** Four-dimensional computed tomography (4DCT) protocol for parathyroid gland.

Parameters	Description
Coverage	Maxilla to carina
Iodinated contrast material administration	100 mL (370 mg iodine/mL) injected at 3–4 mL/s, followed by 40 mL saline flush
Phases	Non-contrast, arterial, delayed venous
Arterial phase	25–30 s after start of injection
Delayed venous phase	60 s after start of injection
Thickness	1.25 mm
Tube voltage (kVp)	140
Tube current (mA)	Minimum 180 and maximum 300
Interval	1 mm
Display field of view (DFOV) (cm)	25

**Table 3:** Difference between multiglandular parathyroid disease, solitary adenoma and carcinoma using clinical and laboratory parameters and imaging.

Clinico-Radio-Pathological Features	Multiglandular Parathyroid Disease	Solitary Parathyroid Adenoma	Parathyroid Carcinoma
Age	Variable, usually >50 years Early onset in hereditary forms	>50 years	Around 50 years Early onset in HPJT and familial forms
Gender	Females	Females	Equal gender incidence
Clinical manifestations	Symptoms of primary hyperparathyroidism and those associated with familial syndromes and MEN (when present)	Asymptomatic Symptoms of primary hyperparathyroidism	Symptoms of primary hyperparathyroidism especially simultaneous bone and renal disease (functional), HPJT, symptoms associated with familial syndromes and MEN (when present), palpable neck nodes, hoarseness of voice due to RLN palsy
Laboratory parameters	Serum calcium: <13 mg/dL Serum PTH: Mildly to severely elevated Hypocalciuria in FHH	Serum calcium: <13 mg/dL Serum PTH: Mildly to moderately elevated	Serum calcium: >14 mg/dL Serum PTH: more than three times upper limit of normal
Imaging features	***Imaging morphology*** Bilaterally enlarged (may be asymmetric) homogeneous polar glands adjacent to thyroid on **US, 4DCT, 4DMRI** ***Imaging characteristics*** Intense homogeneous enhancement on **CEUS**.	***Imaging morphology*** Well circumscribed homogeneous oval/oblong-shaped polar lesion adjacent to thyroid with feeding vessel sign on US and 4DCT. ***Imaging characteristics*** Hypoechoic on **US** Early peripheral enhancement and central washout in the delayed phase on **CEUS.** Hypodense to thyroid on non-contrast scan, usually hyper-enhancing in the arterial phase with feeding vessel sign and washout in the venous phase on **4DCT.** Homogeneous or marbled T2 hyperintensity lesion with fluid fat interface on out-of-phase imaging between thyroid gland and PA, and rapid post-contrast enhancement on **4DMRI.**	***Imaging morphology*** Large (>3 cm) heterogeneous lesion, irregular shape, epicentred adjacent to thyroid with lobulated margins infiltrating thyroid and surrounding structures, short/long-axis ratio >0.76, long axis diameter >30 mm, presence of central and peripheral vascularity, and intratumoural calcification along with metastatic neck nodes on **US, 4DCT, 4DMRI.** Parathyroid carcinoma is differentiated from PA based on morphological features only.
Functional imaging	***Technetium****(**99mTc**) **Sestamibi:*** Focal uptake over bilateral upper and lower poles of thyroid lobes in the early phase, with persistent uptake in the delayed phase.	**Dual-energy CT 4DCT:** Differentiation of parathyroid lesion from thyroid tissue using non-contrast 40-keV virtual monoenergetic images for parathyroid lesions which are isodense to thyroid in the arterial and venous phases. **^99m^Tc *Sestamibi:*** Early phase shows focal increased radiotracer uptake near the superior/inferior polar region of thyroid, with persistent uptake in the delayed phase.	**FDG-PET CT:** Distant metastasis No specific characteristics on Technetium (99mTc) Sestamibi
Pi	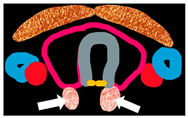	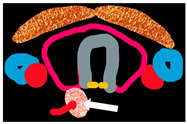	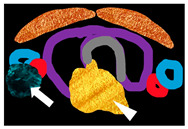

PA: parathyroid adenoma, HPJT: hyperparathyroidism jaw-tumour syndrome. MEN: multiple endocrine neoplasia, RLN: recurrent laryngeal nerve, PTH: primary hyperparathyroidism, FHH: familial hypocalciuric hypercalcaemia, US: ultrasound, CEUS: contrast-enhanced ultrasound, 4DCT: four-dimensional computed tomography, 4DMRI: four-dimensional magnetic resonance imaging, FDG-PET: fluorodeoxyglucose positron emission tomography.

**Table 4:** Advantages and limitations of various imaging modalities for evaluating parathyroid lesions.

Imaging Modality	Advantages	Limitations
US with CEUS	No radiation Easy availability Dynamic scanning technique, hence area of interest can be focused by the operator Low cost Can be performed intraoperatively for guiding the surgeons. CEUS can help differentiate PA from MGD Enlarged lymph nodes associated with a parathyroid lesion may suggest parathyroid carcinoma. Optimal modality for concurrent evaluation of thyroid pathologies.	Operator-dependent. Localisation of ectopic gland difficult and may not be feasible due to limited field of view. Lack of acoustic window limits assessment of lesions in the retroclavicular, mediastinal and retroesophageal locations. Local invasion of structures less well appreciated than with cross-sectional imaging. Superior parathyroid adenomas may be falsely assigned to the inferior position. Reduced sensitivity for depicting far posterior lesions. Reduced effectiveness in obese patients.
^99m^Tc sestamibi or dual tracer ^99m^Tc pertechnetate and ^99m^Tc-sestamibi scintigraphy	Can localise hyperfunctioning parathyroid in orthotopic or ectopic location. Operator-independent Enhanced visualisation of far posterior lesions that US is likely to miss. Both functional and anatomic information with SPECT/CT.	Effective radiation dose of 12 mSv. Superior parathyroid adenomas may be falsely assigned to the inferior position on Technetium-99 sestamibi SPECT/CT. Adenomas with rapid washout can be missed. False positives in presence of thyroid nodules and neck nodes. Reduced sensitivity in those taking calcium channel blockers.
4DCT	Short imaging time High spatial resolution to detect small lesions within the orthotopic and ectopic glands. Features of local invasion and metastatic cervical adenopathy may suggest parathyroid carcinoma. Increased sensitivity for MGD. Improved localisation after failed surgery.	High effective radiation dose of 28 mSv. Need for iodinated contrast media.
4DMRI	No radiation Features of local invasion and metastatic cervical adenopathy may suggest parathyroid carcinoma	Longer scan time Claustrophobic patients unable to tolerate MRI. Need for gadolinium contrast injection.
^18^F-FCH PET	Advantages of both structural and functional information. Can detect small lesions in MGD.	Uptake by generalised neoplastic process, tracer not specific to parathyroid gland. High cost Radiation exposure

US: ultrasound, CEUS: contrast-enhanced ultrasound, SPECT: single photon emission computed tomography, 4DCT: four-dimensional computed tomography, MRI: magnetic resonance imaging, ^18^F-FCH PET: fluoro-choline positron emission tomography, MGD: multiglandular parathyroid disease, PA: parathyroid adenoma.

**Table 5:** Comparative studies evaluating performance of various imaging modalities in primary hyperparathyroidism (PHPT).

Studies	Imaging Modality	PA	MGD	PA and/or MGD (Not Specified)	Comments
Krol et al. [112]	4DCT	*Patient and localisation* S = 70.6% PPV = 86.1% *Lateralization* S = 62.7% PPV = 88.9%	-	-	Significantly higher sensitivity of 4DCT for patient and localisation level
	US + ^99^Tc MIBI SPECT/CT	*Patient and localisation* S = 51.9% 67.9% *Lateralization* S = 44.4% PPV = 85.7%			
Patel et al. [113]	^18^F-FCH PET/CT	-	-	*Patient-wise analysis* S = 92% DR = 92.4% *Lesion-wise analysis* S = 90% *Negative conventional imaging/persistent PHPT* S = 84%	FCH PET/CT scan had a higher pooled sensitivity than 4DCT in detecting patients with PHPT
	4DCT			*Patient-wise analysis* S = 85% DR = 76.85% *Lesion-wise analysis* S = 79% *Negative conventional imaging/persistent PHPT* S = 72%	
He et al. [114]	US	-	-	S = 100%	^99m^Tc-MIBI scintigraphy could increase the specificity in paediatric patients with multigland disease suspected by US.
	^99^Tc MIBI			S = 93.8%	
Christensen et al. [115]	^11^C-Choline PET	-	-	S = 82%	
	Di-SPECT			S = 87%	
Lee et al. [116]	Choline PET-CT	-	-	0.987	Highest surface under the cumulative ranking curve (SUCRA) value of Choline PET-CT for localisation
	MET PET-CT			0.7046	
	MIBI SPECT			0.5465	
	MIBI planar			0.0585	
	Dual tracer			0.3241	
	US			0.1286	
	CT			0.7780	
	MRI			0.4700	
Murugan et al. [117]	4DCT	-	-	S = 96.7% Sp = 66.6% A = 95.2% PPV = 98.3% NPV = 50%	
	4DMRI			S = 96.7% Sp = 66.6% A = 95.2% PPV = 98.31% NPV50%	
de Jong et al. [118]	CT	A = 81%			US and CT could be considered as a first-line imaging modality in patients with PHPT considered for MIP.
	US and CT		A = 50%	S = 88%	
	US and sestamibi	A = 62%	A = 40%	S = 65%	
Whitman et al. [119]	^18^F-FCH PET	S = 0.96			^18^F-FCH PET demonstrates high localisation accuracy in patients with hyperparathyroidism.
	^99m^Tc-sestamibi scans	S = 0.54			
Özdemir et al. [120]	Planar scintigraphy	S = 80.4% Sp = 42.8% PPV = 91.1% A = 75.8%	-	-	
	SPECT/CT	S = 80.4% Sp = 57.7% PPV = 91.1% A = 77.5%			
	US	S = 88.2% Sp = 85.7% PPV = 97.8% A = 87.9%			
	SPECT + US	S = 94.1% Sp = 71.4% PPV = 96% A = 91.3%			
Saerens et al. [121]	US	S = 36/90 40% Sp = 211/221 95.5% PPV = 36/46 78.3% NPV = 211/265 79.6%	-	-	
	Subtraction scintigraphy	S = 24/75 32% Sp = 169/185 91.4% PPV = 24/40 60% NPV = 169/220 76.8%			
	MET-PET/CT	S = 13/22 59.1% Sp = 44/46 95.7% PPV = 13/15 86.7% NPV = 44/53 83%			
	4DCT	S = 5/8 62.5% Sp = 24/25 96% PPV = 5/6 83.3% NPV = 24/27 88.9%			
Bioletto et al. [122]	^18^F-FCH PET	-	-	S = 92% PPV = 95%	Superior performance of 18F-Fluorocholine in terms of sensitivity
	MET-PET			S = 80% PPV = 95%	
Okudan et al. [123]	^99^Tc MIBI SPECT/CT	S = 92.17% PPV = 94.64% A = 87.60%	-	-	Tc-MIBI SPECT/CT is more accurate than ultrasound for the preoperative identification of single PAs in patients with PHPT who are candidates for MIP.
	US	S = 75.89% PPV = 90.43% A = 70.25%			
Kairemo et al. [124]	Dual-phase scintigraphy including SPECT/CT	S = 93%	-	-	
	4DCT	S = 93%			
	US	S = 73%			

PA: parathyroid adenoma, MGD: multiglandular parathyroid disease, US: ultrasound, 4DCT: four-dimensional computed tomography, SPECT: single photon emission computed tomography, MRI: magnetic resonance imaging, ^18^F-FCH PET/CT: fluoro-choline positron emission tomography, A: accuracy, S: sensitivity, Sp: specificity, PPV: positive predictive value, NPV: negative predictive value, DR: detection rate, MIP: minimally invasive parathyroidectomy.
